# Potential pathways for natural active ingredients to intervene in diabetic kidney disease: targeting macrophage infiltration

**DOI:** 10.3389/fphys.2025.1723762

**Published:** 2025-11-24

**Authors:** Yan Yan, Wenru Wang, Yao Chen, Keqin Zhao, Tian Zhan, Xuemin Song, Jiayi Yang, Peng Liu, Renhuan Yu, Gang Wang

**Affiliations:** 1 Hubei University of Chinese Medicine, Wuhan, China; 2 Xiyuan Hospital, China Academy of Chinese Medical Sciences, Beijing, China; 3 Department of Nephrology, Wuhan Hospital of Traditional Chinese Medicine, Wuhan, China

**Keywords:** diabetic kidney disease, macrophage infiltration, inflammation, active compounds, Chinese herbal medicine

## Abstract

Diabetic kidney disease (DKD), a grave microvascular complication of diabetes, is the primary cause of end-stage renal disease. Despite advances in conventional therapies, their limited efficacy underscores the urgent need for novel, multi-target intervention strategies. Macrophage infiltration and the subsequent chronic microinflammation are central to the pathogenesis of renal injury in DKD. A diverse array of natural bioactive compounds are emerging as promising therapeutic agents, capable of modulating these inflammatory pathways. This review investigates the mechanisms underlying the attenuation of DKD progression by six major classes of natural compounds, such as glycosides, diterpenoids, and alkaloids, among others, through the targeting of macrophage infiltration. Collectively, this synthesis offers a compelling case for developing natural product-based, multiple-target strategies to combat DKD. Collectively, this synthesis builds a compelling case for developing multi-target therapeutic strategies derived from natural products to combat DKD.

## 1 Introduction

Diabetic kidney disease (DKD), a significant complication of diabetes involving the microvasculature, is the foremost cause of ESRD worldwide (Liu et al., 2024; Zhao et al., 2024). It is predicted that the global prevalence of diabetes will increase from 643 million in 2030 to 783 million by 2045, with up to 40% of these individuals expected to develop kidney disease. This escalating prevalence positions DKD as a major global health challenge, profoundly impacting patient prognosis and imposing a substantial socioeconomic burden (Hill-Briggs et al., 2020; Pan et al., 2022).

At the core of DKD pathogenesis lies chronic renal microinflammation, hallmarked by the infiltration of macrophages into the glomeruli and tubulointerstitium (Wang et al., 2022). Within the diabetic renal microenvironment, these infiltrated macrophages become activated, releasing a cascade of proinflammatory cytokines and chemokines that drive progressive renal tissue damage and functional decline (Liu et al., 2023; Li et al., 2022). Consequently, targeting macrophage-mediated inflammation represents a critical therapeutic strategy for mitigating DKD progression.

According to guidelines from the American Diabetes Association (ADA) and Kidney Disease: Improving Global Outcomes (KDIGO), the standard treatment for DKD combines renin-angiotensin system inhibitors (ACE inhibitors or angiotensin receptor blockers) with cardiorenal protective medications, including sodium-glucose cotransporter-2 inhibitors (SGLT2i), non-steroidal mineralocorticoid receptor antagonists (ns-MRA), and glucagon-like peptide-1 receptor agonists (GLP-1R). However, this strategy provides only partial renal protection and is associated with various adverse effects (Tang et al., 2021; Zhao et al., 2025; de Boer et al., 2022). This highlights a critical unmet clinical need for novel therapeutic strategies. Natural products, many derived from traditional herbal medicines, are emerging as a promising source of multi-target agents capable of modulating these complex inflammatory pathways (Deng et al., 2025; Wang et al., 2024; Chung et al., 2023). This review, therefore, synthesizes current evidence on the mechanisms by which natural products modulate macrophage infiltration and activity in DKD, providing a rationale for their development as adjunctive therapies to prevent or treat this devastating disease.

## 2 Mechanisms of macrophage infiltration in the intervention of DKD

In adults, the predominant origin of macrophages is from bone marrow hematopoietic stem cells. The development of circulating monocytes that then go on to mature as macrophages is triggered by these progenitors (Chowdhury and Trivedi, 2023). However, certain tissues also harbor self-renewing, embryonically-derived resident macrophage populations (Lazarov et al., 2023). Crucially, the local renal microenvironment dictates macrophage identity and function by inducing tissue-specific gene expression programs that are essential for maintaining homeostasis.

Macrophage infiltration into the kidney is a pivotal early event that drives sustained renal injury in DKD (Jiang et al., 2022). In the context of diabetic conditions, a combination of hyperglycaemia, oxidative stress and advanced glycation end products (AGEs) stimulates renal cells to secrete a range of chemokines and inflammatory mediators. These signals orchestrate the recruitment and subsequent activation of macrophages, thereby initiating and amplifying the local inflammatory cascade (Ansari et al., 2025). Once recruited, these macrophages exhibit remarkable plasticity, polarizing into two distinct functional phenotypes in response to microenvironmental cues, namely, the classically activated (M1) pro-inflammatory and the alternatively activated (M2) anti-inflammatory/reparative subtypes (Wynn and Vannella, 2016). Consequently, inhibiting macrophage infiltration represents a powerful therapeutic approach. This strategy not only limits the expansion of the intrarenal macrophage pool but also modulates the local polarization balance, primarily by reducing the influx of pro-inflammatory monocytes that differentiate into M1-like macrophages. Shifting the balance away from a dominant M1 inflammatory response, which is characterized by citokines such as interleukin (IL)-6 and IL-12, mitigates tissue damage and promotes a microenvironment conducive to M2-mediated repair (Yan et al., 2020). Therefore, a central strategy for modulating the progression of DKD is to target macrophage infiltration.

## 3 Natural medicines modulate macrophage infiltration

**FIGURE 1:**
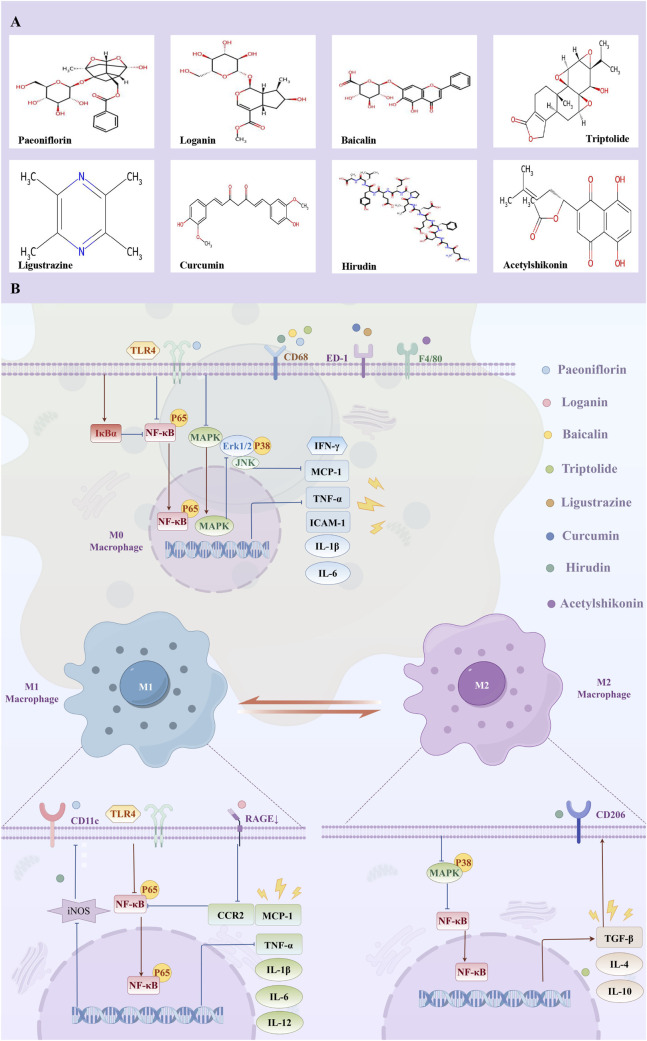
**(A)** Molecular structural formula of natural products; **(B)** The mechanism of natural bioactive compounds attenuating renal macrophage infiltration in diabetic kidney disease.

Numerous bioactive constituents derived from Chinese herbal medicine, such as Paeoniflorin, Loganin, Baicalin, Triptolide, Ligustrazine, Curcumin, Hirudin, and Acetylshikonin, demonstrate protective effects against DKD by mitigating renal macrophage infiltration. They orchestrate this process through a multi-targeted mechanism, concurrently suppressing key signaling pathways including TLR4/NF-κB, RAGE/MCP-1, MAPK, PKC/NF-κB, and TGF-β1/Smad, thereby achieving synergistic renoprotection (Table 1; Figure 1).

**TABLE 1:** The mechanism of natural bioactive compounds attenuating renal macrophage infiltration in diabetic kidney disease.

Compound name	Source	Model	Dosages	Time	*In Vivo/In Vitro*	Mechanism	Effects	References
Paeoniflorin	Paeonia lactiflora Pall	STZ-induced diabetes mice	25,50,100 mg/kg	12W	*In Vivo*	↓CD68,TLR4/NF-κB p65,MCP - 1	↓UA ↓Glomerular mesangial expansion index, tubulointerstitial injuryindex	Shao et al. (2019)
		HG-induced BMDMs	10^−8^,10^−7^,10^−6^,10^−5^,10^−4^ mol/L	24 h	*In Vitro*	↓CD11c,TLR4,iNOS,TNF-α,IL-1β,MCP-1	—	
Loganin	Cornus officinalis	High-fat fed KK/Ay mice	50,100 mg/kg	8W	*In Vivo*	↓iNOS,TNF-α, IL-1β, MCP-1RAGE-MCP-1/CCR2,MCP-1,NF-κB,AGEs, RAGE	↓FBG,24hUTP,BUN,SCr	Du et al. (2021)
		AGEs induced GMCs and RAW264.7	0.1,1.0,10.0 μmol/L	24 h	*In Vitro*	↓iNOS,TLR4,MCP-1/CCR2 signaling pathway	—	
Baicalin	Scutellaria baicalensis Georgi	db/db mice	400 mg/kg	8W	*In Vivo*	↓CD68,IL-1β,IL-6,MCP-1,TNFα,MAPK,Erk1/2, JNK,P38↑Nrf2	↓FBG,24hUTP,ACR,UAER, Glomerular hypertrophy, mesangial matrix expansion	Ma et al. (2021)
Triptolide	*Tripterygium wilfordii Hook f*	STZ-induced diabetes rats	6,12,24 mg/kg	4W	*In Vivo*	↓CD68,IFN-γ,TNFα, NF-κB,MCP-1,TGF-β1 ↑IL-4,IL-10	↓UMA,LYM,FBG,TG,TC	Guo et al. (2016)
Ligustrazine	Ligusticum chuanxiong	STZ-induced diabetes rats	50,150 mg/kg	12W	*In Vivo*	↓ED-1,MCP-1,ICAM-1,NF-κB	↓24hUTP	Wang et al. (2004)
Curcumin	Curcuma longa	STZ-induced diabetes rats	100 mg/kg	8W	*In Vivo*	↓ED-1,NF-κB,IκBα,ICAM-1,MCP-1,TGF-β1,TNF-α,IL-1β	↓24hUTP,FBG,BUN,CrCl ↓Segmental Glomerulosclerosis	Soetikno et al. (2011)
Hirudin	leeches	STZ-induced diabetes rats	5 U/unit/day	8W	*In Vivo*	↓CD68,TNF-α,IL-1β,IL-6,p38 MAPK,NF-κB	↓FBG,24hUTP,BUN,SCr	Han et al. (2020)
		HG-induced MPC5 and PMPs	5 U/mL	48 h	*In Vitro*	↓iNOS,TNF-α,IL-1β,IL-6,p-p38/p38,p-p65/p65,MAPK/NF-κB signaling pathway ↑IL-10, CD206	—	
Acetylshikonin	Lithospermum erythrorhizon	STZ-induced diabetes mice	100 mg/kg	8W	*In Vivo*	↓F4/80,IL-1β, IL-6, MCP-1,ICAM-1	↓BUN,UCr ↓renal fibrosis	Li et al. (2018)

### 3.1 Glycoside compounds

The monoterpene glycoside Paeoniflorin (PF), which is isolated from various *Paeonia* species, is characterized by diverse pharmacological activities. Anti-allergic, antioxidant, and anti-inflammatory activities are among its documented range of effects (Cao et al., 2023). This compound has shown great promise as a treatment for various diseases, including arthritis, psoriasis, lupus and diabetes (Hong et al., 2022). Preclinical evidence strongly supports the utility of PF as a renoprotective compound in DKD (Zhang et al., 2023). Nuclear factor-κB (NF-κB) is a widely distributed nuclear transcription factor that regulates the expression of immune-related genes and participates in innate and adaptive immunity, mediating inflammatory responses (Yu et al., 2020). Toll-like receptors (TLRs) play a pivotal role in the innate immune response, recognising both pathogen- and damage-associated molecular patterns (de Melo et al., 2022). TLRs activate NF-κB transcription through both MyD88-dependent and MyD88-independent pathways, leading to the release of multiple proinflammatory cytokines including tumor necrosis factor alpha (TNF-α), IL-6, and monocyte chemoattractant protein-1 (MCP-1). Among these, the TLR4 signaling pathway plays a pivotal role in the inflammatory response and progressive fibrosis associated with kidney disease (Fock and Parnova, 2021). This activation, through both MyD88-dependent and independent pathways, converges on the transcription factor NF-κB, triggering the expression of proinflammatory cytokines (e.g., TNF-α, IL-6) and the potent chemokine MCP-1. Both experimental TLR4 knockout and clinical observations have confirmed a direct correlation between TLR4 expression, macrophage infiltration, and the severity of renal injury in DKD (Devaraj et al., 2011; Lin et al., 2012). By specifically inhibiting the TLR4/NF-κB p65 signaling axis, PF effectively suppresses this inflammatory cascade, thereby blocking macrophage migration into renal tissue and attenuating the progression of DKD (Shao et al., 2019).

Loganin is the most abundant cycloarene-type triterpene glycoside in *Cornus officinalis* (Zhang et al., 2022). Through its mediation of anti-inflammation, combating of oxidation, and inhibition of fibrosis, it exhibits broad biological activity against various kidney injuries, both acute and chronic (Jiang et al., 2012). Notably, strychnine exhibits renoprotective effects in early-stage DKD (Kong et al., 2023). AGEs are a pathogenic factor in DKD, causing kidney damage through multiple mechanisms (Yuan et al., 2017). AGEs drive renal injury by binding to their receptor (RAGE), which activates downstream signaling cascades, including NF-κB and Protein Kinase C (PKC). The transcription of MCP-1 is subsequently upregulated by this (Nebb et al., 2022). As a key chemokine, MCP-1 orchestrates the recruitment of macrophages to the kidney via its receptor, CC chemokine receptor 2 (CCR2), thus initiating and amplifying the local inflammatory response (He et al., 2023). Investigations into loganin’s mechanism of action highlight its role in inhibiting the RAGE–MCP-1/CCR2 axis as a key mechanism for reducing macrophage infiltration and alleviating renal injury in DKD models. This was substantiated by an *in vivo* study where an 8-week, low-dose treatment significantly improved renal pathology (Du et al., 2021).

Baicalin (BAI) is one of the most abundant flavonoid compounds found in the root of *Scutellaria baicalensis* Georgi (Wen et al., 2023). Modern pharmacological research indicates that BAI has a number of biological properties, such as antitumour, antibacterial, antioxidant and anti-inflammatory effects. This suggests its potential for treating various diseases (Li et al., 2025). In the field of DKD, BAI has been shown to have effective renal protective properties owing to its significant anti-inflammatory and antioxidant effects (Ren et al., 2023). The efficacy of BAI was demonstrated in a trial involving early-stage DKD patients, where it significantly improved markers of renal function, including 24-h urinary protein (UPT) and urinary albumin excretion rate (UAER), by reducing oxidative stress and modulating immune responsiveness (Yang et al., 2019). The mitogen-activated protein kinase (MAPK) signaling pathway is a central regulator of inflammatory responses. Its dysregulated activation prompts the secretion of inflammatory mediators and the infiltration of immune cells into tissues (Moon and Ro, 2021). BAI directly inhibits the phosphorylation of key proteins in the MAPK pathway—extracellular signal-related kinases one and 2 (Erk1/2), C-jun N-terminal kinase (JNK), and p38, thereby blocking downstream inflammatory signaling. Concurrently, it downregulates MCP-1 expression, reducing macrophage chemotaxis (Ma et al., 2021). Concurrently, BAI engages the antioxidant response by activating the nuclear factor E2-related factor 2 (Nrf2) pathway, a master regulator of endogenous antioxidant defenses. By suppressing MAPK-driven inflammation while bolstering Nrf2-mediated cytoprotection, this dual-pronged mechanism synergistically inhibits macrophage infiltration and ameliorates renal damage, as evidenced by reduced albuminuria in DKD animal models (Ma et al., 2021).

### 3.2 Diterpenoid compounds

Triptolide (TP), a diterpene lactone derived from the roots of *Tripterygium wilfordii* Hook. f. has been widely used in the management of inflammatory and immune-mediated diseases due to its significant anti-inflammatory and immunosuppressive properties (Song et al., 2023). It is currently regarded as one of the most exciting bioactive compounds in the shift from traditional to modern medicine (Gao et al., 2021). Recent research demonstrates that TP has a positive effect on multiple kidney diseases, characterised by a significant reduction in proteinuria (He et al., 2015; Zhou et al., 2016; Lv et al., 2023). In DKD, the immunopathology is driven in part by a skewed T-helper (Th) cell balance, favoring pro-inflammatory Th1 and pro-fibrotic Th2 responses (Wu et al., 2011). This inflammatory milieu promotes the NF-κB-dependent upregulation of MCP-1, the principal chemokine orchestrating the kidney’s recruitment of monocytes/macrophages. Local injury is amplified and fibrosis is driven by these recruited immune cells through mechanisms that include the induction of ransforming growth factor β1 (TGF-β1) (Tanase et al., 2022). Preclinical studies in a rat model of DKD confirm that TP mitigates renal injury precisely by targeting this axis: it rebalances the Th1/Th2 ratio, suppresses NF-κB activation, and consequently, inhibits the infiltration of pathogenic monocytes/macrophages (Guo et al., 2016).

Despite its therapeutic promise, the clinical translation of TP is severely hampered by significant systemic toxicity and poor aqueous solubility. To overcome these limitations, next-generation strategies are being pursued. These include the development of structurally optimized analogues with improved safety profiles, such as (5R)-5-Hydroxytriptolide (LLDT-8) and Minnelide, alongside novel drug delivery systems. Such advancements seek to improve the therapeutic index of triptolide-based compounds, with the potential to unlock their use for treating DKD and other inflammatory disorders (Tong et al., 2021).

### 3.3 Alkaloids

Ligustrazine is an efficacious alkaloid monomer extracted from the Chinese medicinal herb *Ligusticum chuanxiong* (Mu et al., 2023). This compound exerts its pharmacological effects through a multi-target mechanism, including improving microcirculatory disorders, inhibiting aldose reductase activity, providing antioxidant and anti-inflammatory effects, inhibiting fibrosis, and regulating autophagy. It demonstrates significant renal protective efficacy in the treatment of DKD (Gong et al., 2023). The clinical efficacy of Ligustrazine is substantiated by a metanalysis of 25 randomized controlled trials involving 1,645 patients. This analysis demonstrated that Ligustrazine significantly improves key markers of renal function, including blood urea nitrogen (BUN) and serum creatinine (SCr), while markedly reducing UTP and UAER. These data establish Ligustrazine as an effective therapy for preserving renal function and mitigating proteinuria in patients with DKD (Wang et al., 2012). The renoprotective mechanism of Ligustrazine is primarily attributed to its ability to suppress tubulointerstitial inflammation, a core pathological feature of DKD driven by macrophage infiltration (Liu S. et al., 2024). Intercellular adhesion molecule-1 (ICAM-1) is a key molecule mediating the adhesion of macrophages to vascular endothelium. Under physiological conditions, it exhibits basal low expression. However, its expression is increased in response to inflammatory stimuli (Ohga et al., 2007). Research indicates that anti-ICAM-1 antibodies effectively inhibit macrophage infiltration into DKD tissues by blocking the function of this molecule. This finding confirms the pivotal role of ICAM-1 in promoting macrophage recruitment in DKD (Wang et al., 2025). Ligustrazine directly targets the upstream signaling cascade governing ICAM-1 expression. Specifically, it inhibits the Protein Kinase C (PKC)/NF-κB signaling axis, which in turn prevents NF-κB-dependent transcription of the *ICAM-172* gene. By downregulating this key adhesion molecule, Ligustrazine effectively diminishes macrophage infiltration into the renal tubulointerstitium, thus alleviating local inflammation and injury (Wang et al., 2004).

### 3.4 Polyphenolic compounds

Turmeric plants are widely cultivated and recognized throughout Southeast Asia, with their rhizomes commonly used as a spice and yellow food coloring (Yu et al., 2019; Chen et al., 2024). Curcumin (CUR) is a polyphenolic compound extracted from *Curcuma longa* L. as its core active ingredient. With its significant antioxidant and anti-inflammatory properties, coupled with favorable pharmacological safety characteristics, it demonstrates considerable therapeutic potential in the management of chronic inflammation, tumors, metabolic disorders and neurological diseases (ALTamimi et al., 2021). Its utility in DKD is substantiated by a meta-analysis of randomized controlled trials, which showed that CUR supplementation not only reduces serum creatinine but also concurrently improves key metabolic and cardiovascular risk factors, including total cholesterol, blood pressure, and fasting blood glucose (Jie et al., 2021). Mechanistically, CUR’s renoprotective effects are largely attributed to its potent inhibition of the NF-κB signaling pathway, a central hub for inflammation and fibrosis in the kidney (Liu et al., 2021). In the quiescent state, NF-κB is sequestered in the cytoplasm by its inhibitor, IκB. Within the diabetic milieu, however, pathogenic stimuli trigger the degradation of IκB, liberating NF-κB for nuclear translocation. Subsequent phosphorylation of its p65 subunit is a critical step that unleashes its transcriptional activity, driving the expression of pro-inflammatory and pro-fibrotic genes (Capece et al., 2022; Wang et al., 2010). Curcumin intervenes at critical nodes within this cascade to suppress the expression of key NF-κB target genes that mediate macrophage infiltration (e.g., *ICAM-1*, *MCP-1*) and fibrosis (*TGF-β1*). By inhibiting this inflammatory-fibrotic axis, CUR effectively reduces immune cell infiltration and extracellular matrix deposition, thereby attenuating the advancement progression of DKD (Soetikno et al., 2011).

### 3.5 Peptide compounds

Hirudin, a polypeptide derived from medicinal leeches, is the most potent natural inhibitor of thrombin known (Tian et al., 2024). While renowned for its clinical use as an anticoagulant, Hirudin’s therapeutic activities extend to anti-inflammatory and anti-fibrotic effects, which are highly relevant to DKD pathology (Liu S-J. et al., 2024). Recent findings have highlighted that thrombin plays a pivotal role in kidney disease and inflammatory regulation. For instance, nanoparticle-specific inhibition of thrombin activity can mitigate ischemia-reperfusion-induced kidney injury and improve renal function (DiSilvestro, 2000). Inflammation and thrombin exhibit bidirectional regulation, jointly driving disease progression. Concurrently, DKD represents a significant microvascular complication of diabetes, with its pathological essence being a chronic inflammatory state (Wang et al., 2023). A central feature of DKD is podocyte injury, which compromises the integrity of the glomerular filtration barrier and leads to proteinuria (Chen et al., 2024). This damage is driven by the pathological activation of intracellular signaling cascades, including the p38 MAPK and NF-κB pathways, which promote apoptosis and the secretion of inflammatory cytokines (Ural et al., 2023; Gong et al., 2021; Cui et al., 2019). Hirudin directly counteracts this pathology. Preclinical evidence demonstrates that Hirudin can suppress macrophage infiltration and proinflammatory cytokine expression through p38 MAPK/NF-κB-dependent mechanisms, thereby alleviating hyperglycemia-induced foot cell apoptosis and inflammatory responses. The functional consequence of this targeted mechanism is a marked improvement in renal function and a significant decrease in proteinuria in DKD animal models (Han et al., 2020).

### 3.6 Naphthoquinone compounds

Acetylshikonin, a naphthoquinone compound isolated from the plant *Lithospermum erythrorhizon*, exhibits pleiotropic biological activities such as antitumor, antimicrobial, and antioxidant effects (Lin et al., 2023a). Its therapeutic potential has been established for cardiovascular diseases, sexual dysfunction, and cancer (Lin et al., 2023b). Notably, Acetylshikonin exerts broad beneficial effects on diabetes and its complications, highlighting its promising potential as a novel treatment for DKD. Activation of the TGF-β1/Smad signaling pathway is closely associated with the fibrotic process in kidney disease. TGF-β1 not only promotes mesangial cell proliferation, mesangial matrix expansion and glomerulosclerosis, but also makes a significant contribution to tubulointerstitial fibrosis, making it a key pathway in the progression of DKD (Wang et al., 2021). Downstream, the Smad protein family, especially Smad2, Smad3, and Smad7, plays a pivotal role in the pathological regulation of DKD. Studies indicate that in STZ-induced DKD models, conditional knockout of Smad2 in fibroblasts using the fibroblast-specific protein-1 (FSP1) promoter significantly reduces renal fibrosis (Loeffler et al., 2018). Additionally, knockout of Smad3 markedly ameliorates pathological features including glomerular basement membrane thickening, extracellular matrix accumulation and proteinuria (Wang et al., 2021). Early renal inflammation and hypertrophy are crucial preliminary stages in the progression of renal fibrosis (Tian et al., 2015). Research in STZ-induced diabetic mouse models has shown that Acetylshikonin significantly alleviates inflammatory responses and fibrotic lesions in the kidneys (Li et al., 2018). Specifically, it inhibits macrophage infiltration and downregulates the expression of inflammatory cytokines IL-1β, IL-6, MCP-1, and the adhesion molecule ICAM-1, thereby effectively reducing local renal inflammation. Trichrome staining further confirmed that Acetylshikonin significantly inhibits renal fibrosis. At the signaling level, Acetylshikonin acts by reducing TGF-β1 levels and Smad2/3 phosphorylation, concurrently restoring Smad7 expression. In summary, Acetylshikonin prevents kidney inflammation and slows down the development of fibrosis in DKD by blocking the TGF-β1/Smad pathway. This results in fewer macrophages infiltrating the kidneys and lower expression of pro-inflammatory factors (Li et al., 2018).

## 4 Conclusion and perspectives

This review synthesizes existing evidence indicating that multiple natural active ingredients converge on the core pathogenic mechanism in DKD, namely, macrophage-mediated inflammation. Although current standard therapies, such as renin-angiotensin system inhibitors, SGLT2 inhibitors, and GLP-1 receptor agonists, provide cardiorenal protection through hemodynamic and metabolic regulation, a substantial residual risk of disease progression persists, primarily driven by ongoing inflammatory and fibrotic processes. The natural compounds discussed herein inhibit macrophage infiltration and downregulate the expression of key inflammatory mediators such as MCP-1 and ICAM-1, thereby intervening in pathological pathways that act synergistically with existing treatments. This offers a promising combinatorial strategy to disrupt the inflammatory-fibrotic axis driving DKD progression.

However, translational challenges remain, as most efficacy data are derived from preclinical models, and issues such as low bioavailability and dose-limiting toxicity hinder clinical application. To realize this synergistic potential, innovation in drug delivery systems and rigorous clinical trials are essential to validate the efficacy and safety of these natural compounds as adjuvants to standard therapy. The ultimate goal is to harness the complementary mechanisms of both conventional and natural agents to achieve superior therapeutic outcomes. Ultimately, this will fulfill their therapeutic value as a multi-target treatment strategy for DKD.
